# *In Silico* Characterization and Virtual Screening of GntR/HutC Family Transcriptional Regulator MoyR: A Potential Monooxygenase Regulator in *Mycobacterium tuberculosis*

**DOI:** 10.3390/biology10121241

**Published:** 2021-11-27

**Authors:** Thanusha Dhananji Abeywickrama, Inoka Chinthana Perera

**Affiliations:** Department of Zoology and Environment Sciences, Faculty of Science, University of Colombo, Colombo 00300, Sri Lanka; thanu@zoology.cmb.ac.lk

**Keywords:** GntR/HutC transcriptional regulators, homology modelling, structure validation, druggability, virtual screening

## Abstract

**Simple Summary:**

In an era where the world faces new diseases and pathogens, another emerging challenge is neglected pathogens becoming more notorious. Transcriptional regulators play a vital role in the pathogenesis and survival of these pathogens. Hence, characterizing transcriptional regulators, either in vitro or *in silico*, is of great importance. Here, we present the first structural characterization of a GntR/HutC regulator in *Mycobacterium tuberculosis* via *in silico* methods. We have suggested its possible role and potential as a drug target as well as identified possible drug leads that can be used for further improvements.

**Abstract:**

*Mycobacterium tuberculosis* is a well-known pathogen due to the emergence of drug resistance associated with it, where transcriptional regulators play a key role in infection, colonization and persistence. The genome of *M. tuberculosis* encodes many transcriptional regulators, and here we report an in-depth *in silico* characterization of a GntR regulator: MoyR, a possible monooxygenase regulator. Homology modelling provided a reliable structure for MoyR, showing homology with a HutC regulator DasR from *Streptomyces coelicolor*. *In silico* physicochemical analysis revealed that MoyR is a cytoplasmic protein with higher thermal stability and higher pI. Four highly probable binding pockets were determined in MoyR and the druggability was higher in the orthosteric binding site consisting of three conserved critical residues: TYR179, ARG223 and GLU234. Two highly conserved leucine residues were identified in the effector-binding region of MoyR and other HutC homologues, suggesting that these two residues can be crucial for structure stability and oligomerization. Virtual screening of drug leads resulted in four drug-like compounds with greater affinity to MoyR with potential inhibitory effects for MoyR. Our findings support that this regulator protein can be valuable as a therapeutic target that can be used for developing drug leads.

## 1. Introduction

Tuberculosis (TB), a disease that has plagued humankind throughout history, is caused mainly by the infection of *Mycobacterium tuberculosis.* It has been hypothesized that the genus *Mycobacterium* originated 150 million years ago, and the modern *M. tuberculosis* strain survived over 70,000 years, claiming millions of lives each year [1,2]. Even though antitubercular chemotherapy is the backbone of TB treatment, deaths due to the emergence of new strains of *M. tuberculosis* that are resistant to some or all antitubercular drugs (multi-drug resistant TB, MDR-TB) currently form a major health problem. Even decades after Koch’s findings, new genetic and molecular insights are still required to divulge the mechanisms involved in the acquisition of drug resistance and the survival of bacteria under stress in the environment. Adaptation to stress responses is primarily mediated through the tight regulation of gene expression, where transcriptional regulators play a fundamental role in the bacterial cell. The genome of *M*. *tuberculosis* encodes more than one hundred putative transcriptional regulators, out of which many need to be characterized.

GntR family of transcriptional regulators constitute one of the most abundant group of proteins among helix-turn-helix regulators distributed throughout the bacterial world. GntR family of proteins are typically two-domain proteins with a DNA binding N-terminal domain (NTD) and a C-terminal effector binding/oligomerization domain (CTD). As characteristic features, NTD has a conserved architecture of winged-helix-turn-helix whereas CTD shows structural heterogeneity, within the family members [3]. Based on the characteristic similarities between the effector binding domain, GntR family is subdivided into six subfamilies: FadR, HutC, MocR, YtrA, AraA, PlmA and DevA [4,5,6].

The HutC subfamily of proteins comprises about 30% of all GntR regulators, the second most abundant family after FadR [7]. The typical structure of HutC regulators consists of an N-terminal HTH domain and a C-terminal ligand-binding/oligomerization domain, which is about 170 amino acids in length [4]. A peculiar feature of the HutC regulators is that they share a commonly conserved effector-binding (EBD) domain, which is the Ubic-like chorismate lyase fold (UTRA) from *E. coli.*, characterized by three short α-helixes and six-stranded antiparallel β-sheets, which forms the core of the structure [8]. HutC/GntR regulators respond to a variety of ligands such as, histidine (HutC) [9], long-chain fatty acids (FarR) [10], trehalose 6-phosphate (TreR) [11], alkylphosphonate (PhnF) [12] and N-Acetylglucosamine-6-phosphate (NagR and DasR) [13,14]. Due to the structural differences among the subfamilies and the variety of ligands they respond to, as well as a lack of characterized HutC regulators, identifying the cognate ligands remains a significant barrier to understanding the function of these regulators. Hence, identifying and characterizing these proteins could provide new insight into their role in bacteria. To this end, we detail in this study the *in silico* characterization of MoyR protein (Rv0792c) from *M. tuberculosis*, annotated as a GntR, and propose possible hit compounds for further validation.

## 2. Materials and Methods

### 2.1. Selection of GntR/HutC Regulators, Multiple Sequence Alignment and Secondary Structure Prediction

Apart from the characterized HutC regulators, other putative GntR/HutC regulators were identified on UniProtKB (https://www.uniprot.org/, accessed on 10 June 2019) and NCBI (https://www.ncbi.nlm.nih.gov/, accessed on 14 June 2019) databases with the aid of previously published data [15,16]. Retrieved sequences were aligned using ClustalW in MEGA-X [17], and GUIDANCE2 server [18] was used to analyze the confidence score of the alignment. This alignment was used to predict the consensus secondary structure arrangement of the regulators including MoyR using the servers, ESPript3 [19] and Jpred4 [20]. HTH domain and the UTRA domains were identified using Simple Modular Architecture Research Tool (SMART) webserver and confidence levels for the prediction were given by E-values. Secondary structure of MoyR was predicted with PDBsum webserver, and the topology map of the monomer was drawn accordingly [21].

### 2.2. Identifying Conserved Residues in C-Terminal Domain of HutC Regulators

As previously mentioned, multiple sequence alignment of HutC regulators was used to analyze the conserved residues in the HutC regulators using WebLogo tool [22].

### 2.3. 3D Structure Modelling and Structure Assessment of the MoyR Model

Homology modelling of MoyR was built using three servers: SWISS-MODEL, Phyre^2^ and I-TASSER [23,24,25]. The quality of the structure was validated using “Verify 3D”, PROCHECK, ProQ, ERRAT and ProSA-web [26,27,28]. Physiochemical parameters of the protein was studied using Expasy’s ProtParam server (www.web.expasy.org/protparam, accessed on 10 June 2019) and the subcellular localization of the MoyR model was predicted using servers Gpos-PLoc, PSORTb, CELLO v.2.5, LoCTree [29,30,31,32].

### 2.4. 3D Structure Modelling, Structure Assessment and Functional Domain Prediction of the Adjacent Gene Encoding Proteins

Homology modelling of the adjacent genes, Rv0791c, Rv0790c, Rv0789c and Rv0793 encoding proteins were done as mentioned above. The structure assessment for all the modelled structures were done in a similar manner as for the MoyR model. Additionally, functional domains of the adjacent genes were identified using NCBI Conserved Domain Database (CDD) and a blastP analysis was carried out using the UniProtKB as the targeted database. Functional domains were identified using matches with more than 70% identity.

### 2.5. Identifying Effector Binding Site and Druggability of MoyR

In order to determine the possible ligand binding pockets, a structure-based and geometry-based prediction was done using metaPocket 2.0 [33]. The metaPocket2.0 server consists of predictors, LIGSITE, PASS, Q-SiteFinder, SURFNET, Fpocket, GHECOM, Concavity and POCASA. The pockets sites identified by the different methods have different ranking scoring functions. In order to make ranking scores comparable a Z-score calculated for each site in different methods and pocket sites of each method were clustered according to their spatial similarity and total Z-score values of a cluster. CavityPlus web server [34] was also used to identify the cavities and the amino acids which the pockets are made of. Binding pockets of DasR, NagR and MoyR were compared using Pocket Match server [35], and amino acids involved in effector recognition were identified using sequence alignment. The conserved residues in the identified binding pocket of MoyR were determined using ConSurf Server. [36]. Druggability of the pockets were identified using PockDrug server [37].

### 2.6. Virtual Screening Study

The modelled structure of MoyR was used to screen the possible hit compounds, and the virtual screening was performed using AutoDock in PyRx virtual screening tool [38]. As for the preliminary screening, a blind docking was carried out where the protein molecule was set to a rigid file while the ligand was moved and rotated to find the best binding modes. Maybridge and ChEMBL were used as chemical databases for screening and approximately 53,000 compounds in total were used. The first 100 compounds with the lowest binding affinity (kcal/mol) were extracted from the docking results. To eliminate false negative values, the ligand interactions were analyzed using Protein–Ligand Interaction Profiler server (https://plip-tool.biotec.tu-dresden.de/plip-web/plip/index, accessed on 24 February 2020) and Discovery Studio Visualizer [39]. Drug-likeness and pharmacokinetics properties of the resulted compounds were determined by SwissADME.

## 3. Results

### 3.1. Secondary Structure of MoyR

**Figure 1:**
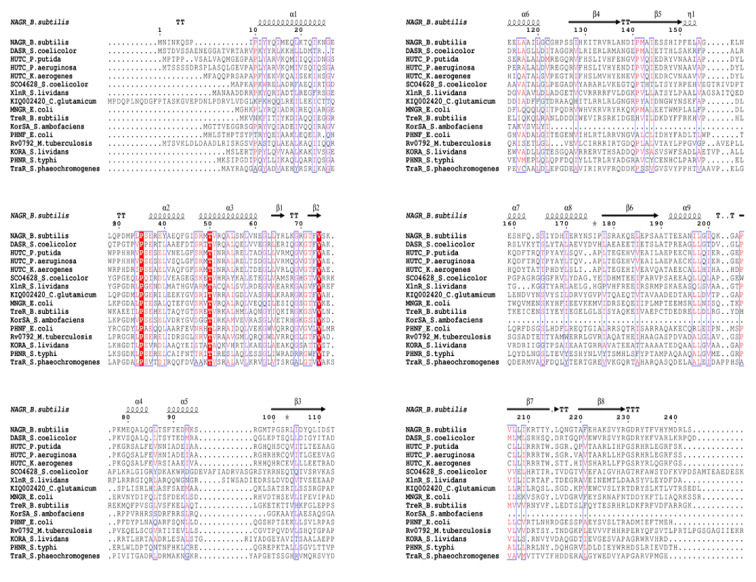
Secondary structure prediction of HutC regulators and MoyR model. Sequence alignment of MoyR and HutC homologs. Conserved residues are highlighted in boxes and secondary structure elements are from the structure of NagR regulator from *Bacillus subtilis*.

**Figure 2:**
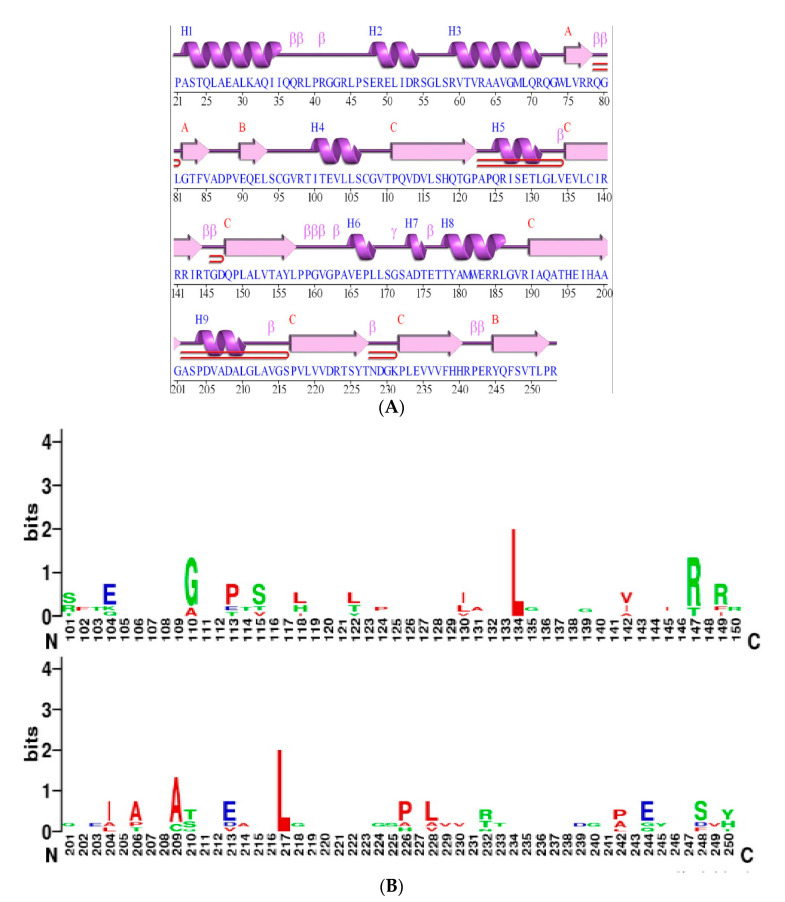
Structure Characteristics of MoyR. (**A**) Secondary structure of modeled MoyR monomer using PDBsum webserver. This consists of nine α-helices and ten β-sheets. (**B**) Analysis of consensus residues of c-terminal effector binding domain of HutC regulators including MoyR. WebLogo represents the relative frequency of nucleotides at corresponding positions.

Multiple sequence alignment of the HutC regulators were carried out by ClustalW, and the confidence level of the multiple sequence alignment was analyzed using GUIDANCE server. The score for the alignment was 0.8434, and all the sequences scored higher than 0.6, which indicates a reliable alignment for further analysis. In the selected HutC regulators, the length of UTRA domain ranged between 128 to 142 amino acids and the length of MoyR UTRA domain was 128 amino acids (106–246 aa) with an E-value of 1.02 × 10^−15^. The length of the DBD of HutC regulators was about 59 amino acids with two highly conserved residues, namely proline and threonine, in the α-helixes (Figure 1). Secondary structure prediction of HutC regulators according to consensus sequences gave a higher number of β-strands towards the c-terminus, which is characteristic to HutC regulators (Figure 1) and the secondary structure prediction of MoyR revealed the same pattern (Figure 2A). The frequency analysis of the bases in the UTRA domain of the selected HutC regulators by WebLogo showed two highly conserved leucine residues at distant positions, in which the height of each base represents the relative frequency at each position (Figure 2B). Two highly conserved leucine residues were found to be at the positions L131 and L210 in MoyR, corresponding to L121 and L202 in TraR protein of *Streptomyces phaeochromogenes* (Figure 1). The L121 residue was identified as a structurally important key residue in the oligomerization and repressor function of TraR [40]. These two leucine residues are conserved in both DasR from *Streptomyces coelicolor* (L130, L208) and NagR from *Bacillus subtilis* (L120, L198), which are MoyR homologues (Figure 1).

### 3.2. Genomic Locus of MoyR

**Figure 3:**
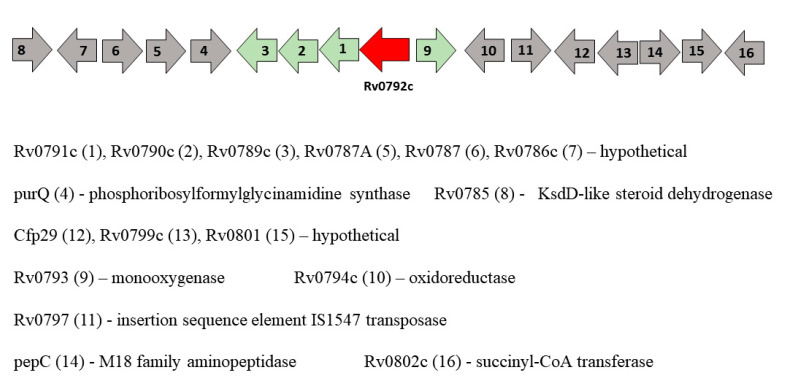
Genomic locus of *moyR* (Rv0792c). Most of the adjacent genes are uncharacterized and code for hypothetical proteins. The genes which can be directly regulated by MoyR protein are highlighted in green.

Many fundamental processes in bacteria, including carbon metabolism [41], amino acid metabolism [9], morphogenesis [42], virulence [43,44], biofilm formation [45], antibiotic resistance [46] and antibiotic production [42], are known to be controlled by GntR regulators. GntR regulators are often located adjacent to the genes that they control, and this could provide an insight into the effectors that these regulator proteins could bind to in the process of regulation. The gene locus of *moyR* consists of many hypothetical proteins (Figure 3). It has been shown that the Rv0789c, Rv0790c, Rv0791c and *moyR* are mostly differentially expressed as an operon in the intracellular environment [47]. According to the correlation catalog of *M. tuberculosis* H37Rv genome, the highest positive correlation with *moyR* was given in Rv0790c and Rv0791c (http://tuberculosis.bu.edu/tbdb_sysbio/CC/Rv0792c.html, accessed on 16 March 2019).

### 3.3. Homology Modelling of MoyR

**Figure 4:**
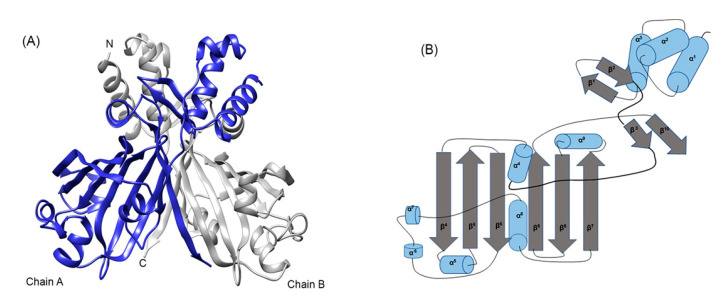
Modeled structure of MoyR. (**A**) Modeled dimeric MoyR protein, each monomer is modeled on chain A and chain B of Moyo respectively. (**B**) Topology diagram of MoyR monomer. Secondary structure elements are displayed as arrows (β-sheets) and cylinders (α-helices). The DBD consist of α-helices, α^1^–α^3^ and β-sheets, β^1^–β^2^. Most of the α-helices (α^5^–α^9^) and β-sheets (β^4^–β^10^) are concentrated in the EBD where the linker segment between the DBD and EBD is highlighted in bold.

The crystal structure of ligand-free HTH type DasR from *Streptomyces coelicolor* (4ZS8) was automatically selected as the template model in all three webservers that were used to model MoyR protein. SWISS-MODEL provided a sequence identity of 30.64% with DasR template and the GMQE value was 0.62 and QMEAN was −1.22. The confidence score for estimating the quality of the predicted model given by C-score in I-TASSER was 0.05, which can be considered as a good confidence score. The TM score and RMSD values were used to measure the structural similarity of the model and the known standard (TM value was 0.72 ± 0.11 and RMSD was 5.5 ± 3.5Å), which indicate a model of correct topology. The backbone confirmation of each residue of the modelled structure was calculated using PROCHECK by analyzing φ/ψ torsion angles [phi (φ) and psi (ψ)] determined by Ramachandran plot.Over 99.8% of the residues were in either the favoured region or the allowed region. Verify 3D further provides a percentage of 73.88 residues with a score of over 0.2 for the MoyR model. The ProQ neural network used for protein quality production in the MoyR model, which gives two scores LGscore and MaxSub. The LG score value was 3.8 (>2.5 very good) and Maxsub was 0.456 (>0.5 very good). The arrangement of different types of atoms with respect to one another in the protein model was assessed by ERRAT, which is sensitive for identifying incorrectly folded regions in preliminary protein models. The overall model quality was assessed by the ProSA-web server, the Z-score value for modelled MoyR is −6.74. The MoyR model was built according to the structural arrangement of DasR regulator. The model of MoyR is a homodimer each consist of two main domains, HTH-DBD and UTRA domain, which is characterized by the six-stranded antiparallel β-sheets in the core of the structure where the effector binding occurs (Figure 4A). The topology of the MoyR monomer was predicted by the PDBsum server, and the topology map was drawn accordingly (Figure 4B). NagR protein from *Bacillus subtilis* also shared a high similarity with the modelled MoyR where both DasR and NagR can be considered as structural homologues of MoyR. Few servers including Gpos-PLoc, PSORTb, CELLO v.2.5 and LoCTree were used to predict the subcellular localization of MoyR, and the cytoplasmic location was predicted with higher confidence values.

### 3.4. Homology Modelling and Functional Annotation of MoyR Adjacent Gene Encoding Proteins

All the *moyR* adjacent gene encoding proteins were modelled, and the structure assessment was carried out as mentioned. The reliability of modelled Rv0790c and Rv0789c were very poor; therefore, these two protein models were excluded from further analysis. Structure modelling of Rv0791c revealed a luciferase-like monooxygenase from *Bacillus cereus* with a sequence identity of 28.79% with a GMQE value of 0.62 and a QMEAN of −2.87. Ramachandran plot analysis of Rv0791c revealed 98.8% of amino acid residues in the favored region and allowed region. Verify 3D analysis results revealed that 88.08% of the residues have a score of more than 0.2 (3D-1D score ≥ 0.2) and Pro-Q analysis yielded a LG score of 5.5, which indicates that the Rv0791c model can be used as a reliable 3D structure for further analysis. Bacterial luciferases are in the class of flavin monooxygenases that catalyze the oxidation of long-chain aldehydes and releases energy in the form of visible light. Even though the crystal structure of Rv0793 is available, the modelled structure was used for docking purposes, which was highly similar to its crystal structure. The amino acid sequences of Rv0789c, Rv0790c, Rv0791c and Rv0793 were used to identify the domains using UniProtKB database. The sequence of Rv0789c did not result in any significant match with a functional domain, whereas blast of Rv0790c resulted in seven hits with more than 75% identity to transglutaminase enzyme from different organisms. The sequence of Rv0791c resulted in a conserved functional domain encoding for an F420 dependent oxidoreductase in four hits with more than 75% identity. Two matches with over 70% identity were identified for Rv0793 corresponding to antibiotic biosynthesis monooxygenases from *Mycobacterium* species and 10 hits with more than 50% identity corresponding to antibiotic biosynthesis binding domains mainly from *Mycobacterium* spp. and *Streptomyces* spp. were also found. The Rv0793 gene encodes a putative monooxygenase which is structurally very similar to *Streptomyces coelicolor* ActVA-Orf6 monooxygenase, which participates in the tailoring of polyketide antibiotic synthesis [48].

### 3.5. Physiochemical Properties of MoyR

MoyR protein monomer consists of 269 amino acids with a molar weight of 28.95 kDa and the theoretical pI is 8.54. A total of 28 negatively charged residues and 30 positively charged residues were identified. The instability index value was 43.8, suggesting that MoyR is unstable outside the cellular environment. The calculated aliphatic index is 101.82, indicating that MoyR is thermally stable, and the GRAVY value (grand average value of hydropathicity) is −0.050 reveals that MoyR is hydrophilic in nature.

### 3.6. Effector Binding Site of MoyR

**Figure 5:**
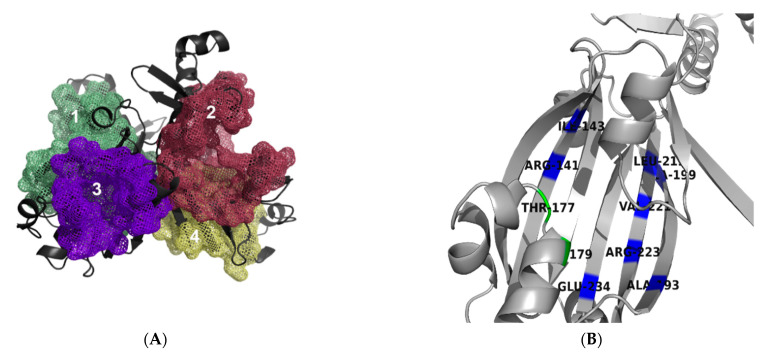
Predicted binding pockets of MoyR. (**A**) Predicted allosites (1 and2) between the DBD-EBD region and the orthosites (3 and 4) in the EBD domain of the dimer. (**B**) Conserved residues of the orthosite in the EBD of a monomer, conserved residues in the β-strands coloured in blue and α-helix residues coloured in green.

MoyR binding pockets were determined using metaPocket 2.0 and CavityPlus web server in which two probable pockets were identified in the region between DBD and EBD (pockets 1 and 2) and two highly probable pockets in the EBD of chain A and B (pockets 3 and 4) (Figure 5A). Hence, pockets 3 and 4 can be considered as the active sites in which ligand binding occurs. Therefore, pockets 1 and 2 can be considered as allosites to which allosteric drugs can bind, whereas pockets 3 and 4 can be considered as orthosites to which orthosteric drugs can bind. These two ligand-binding pockets (pockets 3 and 4) of MoyR and identified NagR and DasR pockets were compared using PocketMatch server. High similarity was obtained in pockets of DasR vs. NagR with a value of 0.8699. Values greater than 0.8 indicate that the pockets are very similar. The value for DasR vs. MoyR pockets was 0.5868 and that for NagR vs. MoyR was 0.6896, suggesting that MoyR shares a pocket similarity to some extent with DasR and NagR. Pairwise sequence identity matrix was generated by Clustal Omega server and overall sequence similarity ranged from 29.15 to 39.17 among the three proteins, indicating high sequence similarity among DasR and NagR. According to the published data, both DasR and NagR respond to the same ligands, glucoseamine-6-phosphate (GlcN-6-P) and N-acetylglucoseamine-6-phosphate (GlcNAc-6-P); for which effector recognition is highly similar [14]. Out of the 16 identified binding site residues in DasR and NagR crystal structure, 12 were similar. When compared with MoyR, only five residues were similar, indicating a lower affinity of glucose moieties to MoyR. To identify the residues that might be conserved in the predicted MoyR binding pocket, a multiple sequence alignment of 150 HutC sequences was generated. Conserved residues of MoyR pocket were identified with the aid of the Consurf analysis server. The identified conserved residues of MoyR pocket are ALA 193, ARG 223, GLU 234, ARG 141, ALA 199, LEU 219, ILE 143 and VAL 221 in the antiparallel β strands and TYR 179 and THR 177 in the α helix (Figure 5B). The identified conserved residues of ligand binding sites of MoyR, DasR and NagR were compared, revealing three highly conserved residues involved in effector binding in all three proteins (Table 1).

### 3.7. Druggability of MoyR and Virtual Screening Analysis

Calculated ligandability and the druggability of the four predicted pockets of MoyR using CavityPlus and PockDrug servers are given in Table 2. Higher druggability of all the predicted four pockets suggests that both allosteric and orthosteric drugs can be used to identify drug leads for MoyR. Overall drug probability of MoyR was calculated and yielded a value of 0.99, suggesting that MoyR has high druggability. Hence, a virtual screening platform was established to screen possible drug candidates for MoyR. The ligands with values lower than −10.0 Kcal/mol were extracted from the virtual screening, and protein–ligand interactions were analyzed. The best four candidate compounds with the lowest binding energy are given in Table 3. The interactions were very similar with all the high-affinity ligands, including conventional hydrogen bonds with highly conserved residues TYR179, ARG223 and GLU234. Many of the predicted binding pocket residues interacted with high affinity ligands via attractive charges, van der Waals bonds, alkyl, Pi-cation and Pi-Pi stacked bonds. Considering the drug-likeness according to the Lipinski rule of five, all the high-affinity compounds can be considered as druglike compounds.

## 4. Discussion

Transcriptional regulators play a crucial role in the survival of bacteria under various stresses and GntR family of HTH-type transcriptional regulators are an important class of proteins in the pathogenesis and survival of bacteria. Even though there are many GntR regulators, in the HutC subfamily, only a few have been crystallized and characterized to date. HutC family members are expected to bind a variety of different effector molecules. Thus far, there is no detailed study that has been carried out on HutC regulators in *M. tuberculosis*. Therefore, this study can be considered a preliminary piece of work, which can provide insights on MoyR structure, its druggability and regulatory role. Amino acid composition itself could provide important information on the structure of a protein as well as its physiochemical parameters. Here, we have identified MoyR as a thermally stable, cytoplasmic protein with a high isoelectric point (pI). Higher pIs contain more electropositive residues on their surfaces and are thus more likely to bind DNA indicative of DNA binding ability of MoyR.

Recent molecular biology studies of *Streptomyces* and *Mycobacterium* have revealed prominent similarities in the developmental and morphological characteristics of the two bacteria. One simple example is the similarities of the two crystal structures, Rv0793 from *M. tuberculosis* and ActVA-Orf6 from *Streptomyces coelicolor*. The protein Rv0793 is predicted as a monooxygenase that participates in the biosynthesis of type II polyketide antibiotics [48]. The *Streptomyces* ActVA-Orf6 monooxygenase is involved in the biosynthesis of actinorhodin produced by type II polyketide synthase (PKSs) [49]. The structural analogue global regulator DasR entailed in signaling cascade from nutrient sensing to development and acts as a switch for antibiotic production in *Streptomyces* [49]. According to the results of this study, functional domain annotation of the *moyR*-adjacent gene encoding proteins are homologous to different monooxygenases. We have previously reported the binding of MoyR to the intergenic region of *Rv0793* and *moyR* [50]. Therefore, this study provides evidence that MoyR has a higher probability of regulating a group of monooxygenases that possibly involves a polyketide antibiotics synthesis or a type II polyketide synthesis pathway in the bacteria. There are no previous reports on isolating antibacterial compounds from *M. tuberculosis* to our knowledge. This finding can be directed towards the probable synthesis of type II polyketides as secondary metabolites. Such antibiotic production would be useful for the bacterium to compete against other bacteria and conquer environmental stresses during survival within the host. We have carried out a preliminary docking study using KEGG pathway intermediates and found that MoyR, Rv0793 and Rv0791c have similar affinities to type II polyketide intermediates (data not shown here). Both the regulators DasR and NagR share numerous effector binding features and respond to the same glucose moieties where MoyR effector binding residues were greatly differing from DasR and NagR, confirming that the affinity for sugar moieties is very weak in MoyR. Hence, by considering the ligand-binding pattern with polyketide intermediates, the genomic locus of *moyR* gene with possible monooxygenases and previously published data [48], it is highly likely that MoyR can play an important role in a polyketide synthesis pathway in the bacteria.

Ligand binding pockets of MoyR were identified using few servers and the key residues were determined according to the conservation of other HutC regulators and structure superimposition with homologue DasR and NagR. As secondary structure prediction and 3D profile further provide information on the spatial arrangement of the amino acids in the protein, this can yield the most probable binding sites for natural ligands and drugs. The conserved TYR 179, ARG 223 and GLU 234 residues in the binding pocket of MoyR are identified as crucial for its function in effector recognition. Two highly conserved leucine residues in the effector binding domain were identified in the sequence alignment of MoyR with other HutC regulators that can be crucial for structure stability and oligomerization of the protein. Binding of the drug-like compounds occurred in the orthosteric site of the effector binding domain of MoyR, indicating that these drug candidates can possibly compete for binding with natural ligands of the MoyR.

The accuracy of a protein model can be assessed by its 3D profile, regardless of whether the model has been derived by X-ray crystallography, NMR spectroscopy or computational methods. The structure assessment data of the 3D model of MoyR provide information on its reliability as a primary screening study of possible ligands. Even though *in silico* characterization would not provide a full picture of the regulatory role of MoyR without supporting biochemical analysis, this study identifies the properties of MoyR and its potential as a drug target. These findings can be extended to study the in vitro binding of the possible natural ligands with MoyR protein and predict its possible role in the cell. The strategies used in this study to annotate the function of MoyR transcriptional regulator and its adjacent genes can be beneficial for designing experimental approaches to further evaluate the function of the genes.

## 5. Conclusions

TB claims millions of lives each year, and the increased emergence of multi-drug-resistant *M. tuberculosis* constitutes a serious global threat. As *M. tuberculosis* has developed resistance to current TB drug regimes, the search for new antibacterial agents directed towards novel targets is of paramount importance. Here, we have identified a GntR/HutC regulator, MoyR involving in regulating a group of monooxygenases. Homology modeling of MoyR and validation of the model suggested that MoyR model can be used as a reliable structure for preliminary screening of drug compounds. The high druggability of MoyR indicates that this protein could be useful as a drug target, and we have identified the best hit compounds for MoyR that warrant further validation using in vitro work.

## Figures and Tables

**Table 1:** Identified conserved residues in binding pockets of MoyR, DasR and NagR involve in effector binding. Highly conserved residues are in bold.

MoyR.	DasR	NagR
ARG 141	ARG 142	ARG 133
THR 177	SER 175	SER 165
**TYR 179**	**TYR 177**	**TYR 167**
VAL 221	LEU 219	ILE 209
**ARG 223**	**ARG 212**	**ARG 211**
**GLU 234**	**GLU 232**	**GLU 222**

**Table 2:** Estimated druggability of the predicted binding pockets of MoyR using CavityPlus and PockDrug servers. In ligandability Pred. Max pK_d_ value greater than six suggests that all the cavities are suitable as binding sites. Drug score is calculated on the basis of the binding structure alone by using a desolvation-based free energy model.

	CavityPlus Server		PockDrug Server
	Ligandability Pred. Max pK_d_	Drug score	Druggability
Cavity 1	9.31	3129	0.92
Cavity 2	11.34	2694	0.93
Cavity 3	11.35	1445	0.91
Cavity 4	10.24	1014	0.94

**Table 3:** Best four lead compounds with high affinity of binding with MoyR.

Compound.	Molecular Formula	Binding Energy (kcal/mol)	Interacting Residues in Binding Pockets 3 and 4
3-(4-fluorobenzyl)-4-methyl-2-oxo-2H-chromen-7-yl 3-(trifluoromethyl)benzene-1-sulfonate	C_27_H_23_ F_3_ N_4_ O_4_	−11.1	VAL113, PRO111, ILE100, VAL 97, VAL153, VAL236, HIS240, THR177, THR101, PHE 238, ARG 223
N-[3-[3-[(phenylsulfonyl)amino]-5-(trifluoromethyl)benzyl]-5-(trifluoromethyl)phenyl]benzenesulfonamide	C_27_H_20_F_6_N_2_O_4_ S_2_	−11	ARG98, VAL221, VAL153, ALA155, VAL113, ARG141 THR101, TYR179,ARG223, PHE238, ILE139, GLU176, ALA173
N’1-[3-(trifluoromethyl)benzoyl]-2-[2,6-dimethyl-4-(3-methyl-4-oxo-3,4-dihydrophthalazin-1-yl)phenoxy]ethanohydrazide	C_27_H_23_F_3_N_4_ O_4_	−10.9	PRO111, VAL113, VAL236, VAL153, ILE100, TYR179, ALA 173, HIS240, ARG223, GLU234, PHE238, GLU92
CHEMBL3222137—name undefined	C_29_H_27_NO_8_	−10.6	VAL 221, HIS 195, VAL 97, VAL 153, VAL236, ILE100, ALA173, TYR179, THR101, GLN 112, VAL113

## Data Availability

Not applicable.

## References

[1] Gradmann C. (2001). Robert Koch and the Pressures of Scientific Research: Tuberculosis and Tuberculin. Med. Hist..

[2] Keshavjee S., Farmer P.E. (2012). Tuberculosis, drug resistance, and the history of modern medicine. N. Engl. J. Med..

[3] Jain D. (2015). Allosteric control of transcription in GntR family of transcription regulators: A structural overview. IUBMB Life.

[4] Rigali S., Derouaux A., Giannotta F., Dusart J. (2002). Subdivision of the helix-turn-helix GntR family of bacterial regulators in the FadR, HutC, MocR, and YtrA subfamilies. J. Biol. Chem..

[5] Lee M.H., Scherer M., Rigali S., Golden J.W. (2003). PlmA, a new member of the GntR family, has plasmid maintenance functions in Anabaena sp. strain PCC 7120. J. Bacteriol..

[6] Hoskisson P.A., Rigali S., Fowler K., Findlay K.C., Buttner M.J. (2006). DevA, a GntR-like transcriptional regulator required for development in Streptomyces coelicolor. J. Bacteriol..

[7] Hoskisson P.A., Rigali S. (2009). Variation in Form and Function, The Helix-Turn-Helix Regulators of the GntR Superfamily. Advances in Applied Microbiology.

[8] Aravind L., Anantharaman V. (2003). HutC/FarR-like bacterial transcription factors of the GntR family contain a small molecule-binding domain of the chorismate lyase fold. FEMS Microbiol. Lett..

[9] Allison S.L., Phillips A.T. (1990). Nucleotide sequence of the gene encoding the repressor for the histidine utilization genes of Pseudomonas putida. J. Bacteriol..

[10] Quail M.A., Dempsey C.E., Guest J.R. (1994). Identification of a fatty acyl responsive regulator (FarR) in Escherichia coli. FEBS Lett..

[11] Schöck F., Dahl M.K. (1996). Expression of the tre operon of Bacillus subtilis 168 is regulated by the repressor TreR. J. Bacteriol..

[12] Gebhard S., Busby J.N., Fritz G., Moreland N.J., Cook G.M., Lott J.S., Baker E.N., Money V.A. (2014). Crystal structure of PhnF, a GntR-family transcriptional regulator of phosphate transport in Mycobacterium smegmatis. J. Bacteriol..

[13] Resch M., Schiltz E., Titgemeyer F., Muller Y.A. (2010). Insight into the induction mechanism of the GntR/HutC bacterial transcription regulator YvoA. Nucleic Acids Res..

[14] Fillenberg S.B., Friess M.D., Korner S., Böckmann R.A., Muller Y.A. (2016). Crystal structures of the global regulator DasR from streptomyces coelicolor: Implications for the allosteric regulation of GntR/HutC Repressors. PLoS ONE.

[15] Vindal V., Ranjan S., Ranjan A. (2007). *In silico* analysis and characterization of GntR family of regulators from Mycobacterium tuberculosis. Tuberculosis.

[16] Suvorova I.A., Korostelev Y.D., Gelfand M.S. (2015). GntR Family of Bacterial Transcription Factors and Their DNA Binding Motifs: Structure, Positioning and Co-Evolution. PLoS ONE.

[17] Kumar S., Stecher G., Li M., Knyaz C., Tamura K. (2018). MEGA X: Molecular evolutionary genetics analysis across computing platforms. Mol. Biol. Evol..

[18] Sela I., Ashkenazy H., Katoh K., Pupko T. (2015). GUIDANCE2: Accurate detection of unreliable alignment regions accounting for the uncertainty of multiple parameters. Nucleic Acids Res..

[19] Robert X., Gouet P. (2014). Deciphering key features in protein structures with the new ENDscript server. Nucleic Acids Res..

[20] Drozdetskiy A., Cole C., Procter J., Barton G.J. (2015). JPred4: A protein secondary structure prediction server. Nucleic Acids Res..

[21] Laskowski R.A., Hutchinson E.G., Michie A.D., Wallace A.C., Jones M.L., Thornton J.M. (1997). PDBsum: A web-based database of summaries and analyses of all PDB structures. Trends Biochem. Sci..

[22] Schneider T.D., Stephens R.M. (1990). Sequence logos: A new way to display consensus sequences. Nucleic Acids Res..

[23] Waterhouse A., Bertoni M., Bienert S., Studer G., Tauriello G., Gumienny R., Heer F.T., De Beer T.A.P., Rempfer C., Bordoli L. (2018). SWISS-MODEL: Homology modelling of protein structures and complexes. Nucleic Acids Res..

[24] Kelley L.A., Mezulis S., Yates C.M., Wass M.N., Sternberg M.J.E. (2015). The Phyre2 web portal for protein modeling, prediction and analysis. Nat. Protoc..

[25] Zhang Y. (2008). I-TASSER server for protein 3D structure prediction. BMC Bioinform..

[26] Bowie J.U., Luthy R., Eisenberg D. (1991). A Method to Identify Protein Sequences That Fold into a Known Three-Dimentional Structure. Science.

[27] Lüthy R., Bowie J.U., Eisenberg D. (1992). Assessment of protein models with three-dimentional profiles. Nature.

[28] Wiederstein M., Sippl M.J. (2007). ProSA-web: Interactive web service for the recognition of errors in three-dimensional structures of proteins. Nucleic Acids Res..

[29] Shen H.-B., Chou K.-C. (2007). Gpos-PLoc: An ensemble classifier for predicting subcellular localization of Gram-positive bacterial proteins. Protein Eng. Des. Sel..

[30] Yu N.Y., Wagner J.R., Laird M.R., Melli G., Rey S., Lo R., Dao P., Cenk Sahinalp S., Ester M., Foster L.J. (2010). PSORTb 3.0: Improved protein subcellular localization prediction with refined localization subcategories and predictive capabilities for all prokaryotes. Bioinformatics.

[31] Yu C.-S., Chen Y.-C., Lu C.-H., Hwang J.-K. (2006). Prediction of Protein Subcellular Localization. PROTEINS Struct. Funct. Bioinform..

[32] Nair R., Rost B. (2005). Mimicking cellular sorting improves prediction of subcellular localization. J. Mol. Biol..

[33] Zhang Z., Li Y., Lin B., Schroeder M., Huang B. (2011). Identification of cavities on protein surface using multiple computational approaches for drug binding site prediction. Bioinformatics.

[34] Xu Y., Wang S., Hu Q., Gao S., Ma X., Zhang W., Shen Y., Chen F., Lai L., Pei J. (2018). CavityPlus: A web server for protein cavity detection with pharmacophore modelling, allosteric site identification and covalent ligand binding ability prediction. Nucleic Acids Res..

[35] Nagarajan D., Chandra N. PocketMatch (version 2.0): A parallel algorithm for the detection of structural similarities between protein ligand binding-sites. Proceedings of the 2013 National Conference on Parallel Computing Technologies (PARCOMPTECH).

[36] Ashkenazy H., Abadi S., Martz E., Chay O., Mayrose I., Pupko T., Ben-Tal N. (2016). ConSurf 2016: An improved methodology to estimate and visualize evolutionary conservation in macromolecules. Nucleic Acids Res..

[37] Hussein H.A., Borrel A., Geneix C., Petitjean M., Regad L., Camproux A.C. (2015). PockDrug-Server: A new web server for predicting pocket druggability on holo and apo proteins. Nucleic Acids Res..

[38] Dallakyan S., Olson A.J. (2015). Small-Molecula Library Screening by Docking with PyRx. Methods Mol. Biol..

[39] (2010). Accelrys Discovery Studio Visualizer v 3.5.

[40] Kataoka M., Tanaka T., Kohno T., Kajiyama Y. (2008). The carboxyl-terminal domain of TraR, a Streptomyces HutC family repressor, functions in oligomerization. J. Bacteriol..

[41] Titgemeyer F., Reizer J., Reizer A., Tang J., Parr T.R., Saier M.H. (1995). Nucleotide sequence of the region between crr and cysM in Salmonella typhimurium: Five novel ORFs including one encoding a putative transcriptional regulator of the phosphotransferase system. DNA Seq..

[42] Hillerich B., Westpheling J. (2006). A new GntR family transcriptional regulator in Streptomyces coelicolor is required for morphogenesis and antibiotic production and controls transcription of an ABC transporter in response to carbon source. J. Bacteriol..

[43] Wu K., Xu H., Zheng Y., Wang L., Zhang X., Yin Y. (2016). CpsR, a GntR family regulator, transcriptionally regulates capsular polysaccharide biosynthesis and governs bacterial virulence in Streptococcus pneumoniae. Sci. Rep..

[44] Casali N., White A.M., Riley L.W. (2006). Regulation of the Mycobacterium tuberculosis mce1 operon. J. Bacteriol..

[45] Lord D.M., Uzgoren Baran A., Soo V.W.C., Wood T.K., Peti W., Page R. (2014). McbR/YncC: Implications for the Mechanism of Ligand and DNA Binding by a Bacterial GntR Transcriptional Regulator Involved in Biofilm Formation. Biochemistry.

[46] Truong-bolduc Q.C., Hooper D.C. (2007). The Transcriptional Regulators NorG and MgrA Modulate Resistance to both Quinolones and beta-Lactams in Staphylococcus aureus. J. Bacteriol..

[47] Arun P.V.P.S., Miryala S.K., Rana A., Kurukuti S. (2018). System-wide coordinates of higher order functions in host-pathogen environment upon Mycobacterium tuberculosis infection. Sci. Rep..

[48] Lemieux M.J., Ference C., Cherney M.M., Wang M., Garen C., James M.N.G. (2005). The crystal structure of Rv0793, a hypothetical monooxygenase from M. tuberculosis. J. Struct. Funct. Genomics.

[49] Rigali S., Titgemeyer F., Barends S., Mulder S., Thomae A.W., Hopwood D.A., van Wezel G.P. (2008). Feast or famine: The global regulator DasR links nutrient stress to antibiotic production by Streptomyces. EMBO Rep..

[50] Abeywickrama T.D., Perera I.C. RV0792c; a potential drug target for Mycobacterium tuberculosis. Proceedings of the Seventh International Conference on Advances in Applied Science and Environmental Technology-ASET 2017.

